# Pyrolysis Kinetics and Flammability Evaluation of Rigid Polyurethane with Different Isocyanate Content

**DOI:** 10.3390/molecules26082386

**Published:** 2021-04-20

**Authors:** Lin Jiang, Filippo Berto, Dan Zhang

**Affiliations:** 1 School of Mechanical Engineering, Nanjing University of Science and Technology, Nanjing 210094, China; ljiang@njust.edu.cn; 2 Department of Mechanical and Industrial Engineering, Norwegian University of Science and Technology (NTNU), NO-7491 Trondheim, Norway; filippo.berto@ntnu.no; 3 School of Chemical Engineering, Nanjing University of Science and Technology, Nanjing 210094, China

**Keywords:** genetic algorithm, polyurethane, isocyanate content, pyrolysis

## Abstract

Polyurethane (PU) is a typical product of the reaction between isocyanate and polyol, whose ratio would greatly influence material properties. In this paper, to investigate the influence of isocyanate on PU thermal stability and flammability, three kinds of rigid polyurethanes (RPUs) with different isocyanate ratio (1.05, 1.1, and 2.0) were manufactured in a laboratory and employed to have a series of TG (thermogravimetry), DSC (differential scanning calorimetry), and cone calorimetry tests. Kissinger’s method was used to calculate the activation energy and judge their stabilities. However, for such a complex degradation which consists of five reactions, it does not make sense by Kissinger method to obtain only two peak active energies. Considering complexity of PU degradation in air, genetic algorithm (GA) was employed to calculate kinetic triplets of five sub-reactions. The effects of isocyanate contents on each sub-reaction stability were obtained and then analyzed. By cone calorimeter testing, we found that great differences in heat release rate data. However, DSC analysis showed a complete opposite changed trend. Such difference is caused by DSC and calorimeter’s sample morphology, the former using grinded polyurethane powders but the latter polyurethane foam block.

## 1. Introduction

Polyurethanes (PUs) have been found using in a growing number of applications in building constructions for decades, such as furniture, appliances, and thermal insulation exterior walls, making them the most versatile plastic materials. Polyurethane (PU) is a typical product of the reaction between isocyanates and polyols [1]. The repeating unit in PU is the urethane linkage produced from the reaction of an isocyanate –NCO with an alcohol –OH. Rigid PU (RPU) is one of the most common forms in our life, which is usually used as thermal insulation materials in building construction. The traditional synthesis method for RPU is usually prepared by using isocyanates and polyols as reactants.

As a widely used thermal insulation material, RPU performs a large fire hazardous behavior [2,3]. For example, the fire disaster that occurred at a high-rising building in Shanghai Jing’an District resulted in more than 120 people dead and injured. The investigation of this shocking fire disaster showed that flame caused by RPU in exterior walls could propagate quickly from the ignition point to the whole building. It is widely accepted that thermal degradation is the first step of the material burning process [4]. Thus, degradation study of building-used construction materials is of great significance to realize its hazard and to prevent and control building fire disasters [2,3,4].

The degradation mechanism of PU has been extensively studied by other researchers. Pyrolysis-gas chromatography/mass spectrometry (Py-GC/MS) and thermogravimetry (TG) are commonly used to investigate its degradation mechanism. R Font et al. [4] took experiments to study the kinetics of PU degradation and the evolution of gaseous volatiles using TG. Zhang et al. [5] studied the pyrolysis of synthesized PU and identified more than 20 characteristic volatile pyrolyzates by on-line MS, which reflects the structure and pyrolysis mechanisms. A relatively detailed review was given by Chattopadhyay [6] PU the thermal decomposition and relative flame retardant. Jiao et al. [7] investigated the degradation mechanism of PU in the nitrogen atmosphere, confirming the temperature ranges of escaped isocyanates and polyols. Meanwhile, Jiao et al. [8] also studied PU kinetics degradation and its pyrolysis volatiles. Using the exact PU degradation mechanism, Jiang et al. [9] developed a kinetic model about PU degradation in nitrogen, which could be used to calculate mass variation of reactants, intermediates, and products. Then its accuracy was verified by TG-mass spectrometry (TG-MS) and Fourier transform infrared (FTIR).

It has been concluded that the increase of isocyanate content in PU could influence the onset decomposition temperature and change the side product formation. The excess of isocyanate in the reaction leads to side reactions generating allophanate and biuret. The allophanate and biuret crosslinks decompose quite readily by heating, whereas the conventional urethane and urea crosslinks decompose at relatively higher temperatures. What is more, isocyanates could generate uretidione (dimer) and isocyanurate (trimer) with the way of addition polymerization [1].

When this research was begun, although large amounts of research have been reported on the mechanism and chemical kinetic of PU degradation, there were, to the knowledge of the authors, few attentions have been paid on the thermal stability study of RPU with different isocyanate index. Isocyanate index of PUs around the market maintains 1.1. Usually, the isocyanate index of RPU is larger than 1.0. When we manufactured polyurethane with isocyanate index 1.5, we found that the thermal stability showed few differences with that of isocyanate index 1.1. Taken together, to ensure that the form of PU is rigid (>1.1) and different isocyanate index gradients are needed, three kinds of PUs with different isocyanate ratios, 1.05, 1.1, and 2.0, were manufactured. In the following paper, we call them P105, P110, and P200 for short, respectively. The thermal degradation kinetics in the air environment was investigated in detail by three single heating rates (10, 15, and 20 °C min^−1^) using TG.

For comparison, the Kissinger method [10] was used to calculate their activation energies of three PUs. Considering that the Kissinger method could only obtain the activation energies at two peak temperatures, however, five sub-reactions exist in fact, so a novel optimization method was employed to obtain the kinetic triplet of each reaction, which was called genetic algorithm (GA) [9,10,11,12,13,14]. Then thermal stability of each step reaction could be compared for different PUs.

## 2. Materials, Experiments, and Models

### 2.1. Raw Materials

Polyether polyol with a hydroxyl value of 454.9 mg KOH/g was produced by China National Chemical Co. Ltd. Polymeric 4,4-diphenylmethane diisocyanate (MDI) was obtained from BASF Corp. Triethylene diamine, as an amine catalyst, was dissolved in diethylene glycol with 33%. Dibutyltin dilaurate, as a tin catalyst, was provided by Air Products Corp. of China. The formula used in this study was typical for building-used thermal insulation systems in the materials market. The components used in the preparation of PUs are presented in Table 1.

### 2.2. Preparation of PU

All PUs in this paper were synthesized by a one-step method. Briefly, the mixture of catalysts, surfactants, water, and polyether polyol was poured into a cup, followed by adding an appropriate quantity of MDI. Then, the mixture was stirred by a blender and poured into a 200 × 100 × 80 mm^3^ mold with a detachable lid to produce free-rise foam. The foaming procedure lasted approximately one and a half minutes. Afterward, the new PU was set for 24 h at 70 °C in the incubator before test.

### 2.3. TG and DSC

TG testing was carried out on SDT Q600 (TA Instruments, New Castle, DE, USA). A sample of ground PU powder with 6.0 mg was put into an aluminum oxide crucible. The temperature ran from 25 to 800 °C with three heating rates (10, 15, and 20 °C min^−1^) in the air atmosphere with a purge airflow of 100 mL min^−1^.

### 2.4. Cone Calorimeter

The cone calorimeter (Stanton Redcroft, UK) test was performed according to ISO 5660 standard procedures. Each specimen of dimensions 100 × 100 × 20 mm was wrapped in aluminum foil and exposed horizontally to an external heat flux of 35 kW/m^2^.

### 2.5. Kinetic Method and Kissinger Method

In polymer degradation TG study, mass loss rate (MLR) could be expressed by the arithmetic product of two-part functions [13,14,15,16]. One is about heating temperature, and the other is reactive conversion percent. Then MLR could be written to:

d*α*/d*t* = β(d*α*/d*T*) = k(*T*)f(*α*) (1)

where *α* is conversion percent, *t* is time, β is heating rate, and *T* is temperature. k(*T*) is chemical reaction rate, which could be described by Arrhenius law, so Equation (1) could be expressed furtherly by:

d*α*/d*t* = *A*exp[−*E*/(R*T*)]f(*α*) (2)

where *E* is activation energy, and *A* is a pre-exponential factor. By performing kinetic studies, significant scientific details regarding the thermal stability and decomposition mechanism could be obtained by the analysis of kinetic triplets, i.e., activation energy, reaction order, and pre-exponential factor [17,18]. In the following paper, we would introduce two kinetic methods, the Kissinger method and the GA method.

Kissinger method is an isoconversional kinetic method that is based on the following expression:

ln(*β*/*T*_p_^2^) = ln(R*A*/*E*) − *E*/(R*T*_P_) (3)

where *β* is the heating rate and TP means the peak temperature of derivative thermogravimetry (DTG). A straight line could be obtained when ln(*β*/*T*_P_^2^) is plotted against 1/*T*_P_, by which the activation energy could be obtained.

### 2.6. An Introduction to GA Method

GA was developed from the base of Darwin’s evolutionism [11]. It has been applied in many areas to solve multi-dimensional parameter optimization [12,13,14]. Meanwhile, it has also been applied to the optimization of chemical reaction mechanisms and kinetic triplet calculation. A corresponding introduction about GA would be introduced.

The operation of GA is initialized by the creation of a new generation. Each group of parameters in the generation is called individual. The parameter in the individual is defined as a gene. In a chemical dynamic solution problem, one reaction corresponds to one set of triplets. In addition, each triplet is just one individual. All individuals could make up one population. Gene value of one individual is determined by the following equation:

$$g_{j}^{i}\;=\;g_{j,\min}^{i}\;+\;r(g_{j,\max}^{i}\;-\;g_{j,\min}^{i})$$ (4)

where $g_{j,\max}^{i}$ and $g_{j,\min}^{i}$ are the upper and lower bounds of the *i*-th gene, the *j*-th individual. In addition, r is a random number distributed from 0 to 1.

Differences between experimental results and numerical calculations are defined as fitness. For each individual, fitness could be used to judge being eliminated or preserved. The higher fitness means that the individual values are closer to theoretical ones, and these individuals should be preserved. On the contrary, lower-fitness individuals would then be eliminated. In the degradation kinetics area, the fitness of individuals in one generation is calculated as:

*ϕ* = a(∑|*MLR*_exp_ − *MLR*_cal_|)^−1^ + (1 − a)(∑|*m*_exp_ − *m*_cal_|)^−1^ (5)

where a is regarded as a mass coefficient, indicating a mass factor to fitness between mass and MLR.

Crossover is a message exchanging process during offspring reproduction. When individuals are selected to crossover as parents, a new generation would be produced by linear combinations of parents. If not selected, genes in parents should be duplicated directly to offspring. In order to avoid optimal local solution, new offspring should be mutated. The probability of mutation is very small, which is 0.05 in this paper. Once mutation occurred, the parameter would begin to change in the range of upper and lower bounds.

## 3. Results and Discussions

### 3.1. Thermal Behaviors

**Figure 1:**
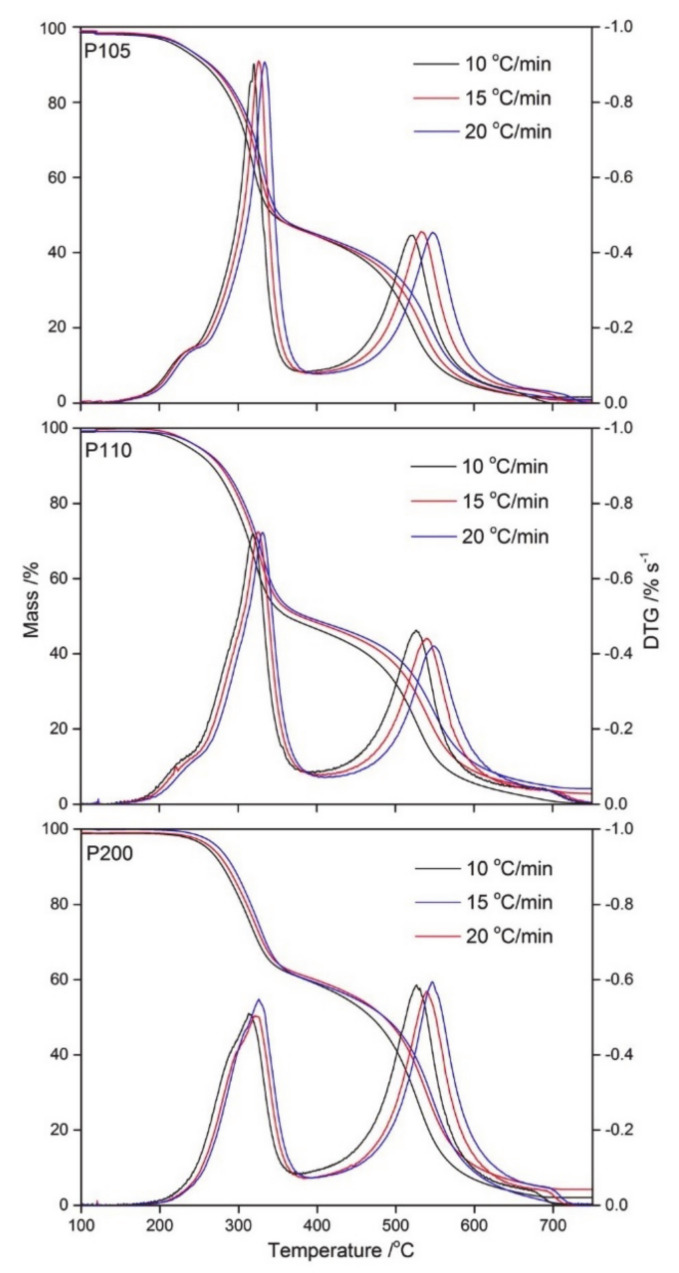
TG and DTG curves of P105, 110, and 200 in the air at 10, 15, and 20 °C min^−1^.

TG and DTG curves of PU samples in the air at 10, 15, and 20 °C min^−1^ were shown in Figure 1. Compared with test results in the nitrogen atmosphere [7,8], we could find that two main mass loss stages occur during degradation in air, while only one mass loss stage could be found in nitrogen [7,8]. Figure 1 shows that TG curves of three PUs’ degradation processes in the air have the same variation tendencies, which could be separated into two phases visually. This is due to their same PU degradation process, just the difference in isocyanate ratio. The fundamental structural unit in PU is urethane [1]; meanwhile, PU has a hard segment and a soft segment. Some unreacted isocyanate would also exist in RPU.

**Figure 2:**
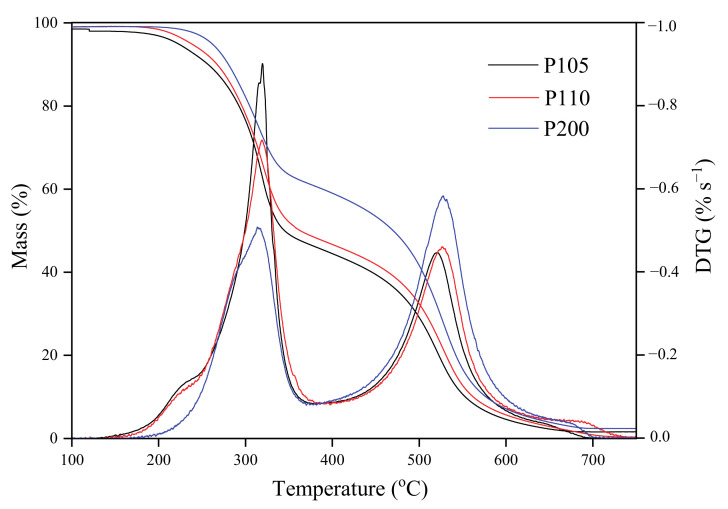
TG and DTG curves of P105, 110, and 200 in the air at 10 °C min^−1^.

Figure 2 is TG and mass variations of P105, P110, and P200 at 10 °C min^−1^ in the air atmosphere, which would be used to analyze the effects of isocyanate contents on PU degradation. As shown in Figure 2 and Table 2, the first-stage degraded temperature would increase greatly with raising isocyanate contents. What is more, the mass loss of the first stage would decrease, and the second stage, the oxidative part, would increase. However, the variation of isocyanate content makes few differences to the initial degradation temperature for second peak visually from Figure 2. The peak temperatures drawn from Figure 2 would be used to calculate activation energy in the following paper.

### 3.2. Kinetic Analysis

The active energies at two peak temperatures could be obtained when ln(β/T_P_^2^) is plotted against 1/*T*_P_. As Table 3 shows in degradation and oxidation phases, although P105 had the lowest isocyanate index, it has the highest activation energy values. In real RPU production, P110 is the most commonly manufactured type considering material cost and molding, but it has the lowest activation energy with an average of 33% lower than P105, which means P105 is much more stable and difficult to degrade than P110 when heating. With an increase in isocyanate index upper than 1.10, the activation energy of P200 would increase but not satisfactorily.

Actually, the degradation of PU in the air could be divided into several steps according to its degradation mechanism, instead of two steps shown by TG curves. Kissinger method could only provide two activation energies in peak temperatures, which is much inconsistent with actual degradation process. Researches by previous scholar [1,8] about PU degradation process in air/nitrogen atmosphere has been obtained a lot. In this paper, considering previous PU degradation mechanisms, five reactions would be applied to activation energy calculation and the creation of degradation modeling.

According to the above descriptions of PU degradation, we propose here a degradation mechanism of PU in air, including isocyanates pyrolysis, urethane bond (UB) pyrolysis, isocyanate segments (ISs) pyrolysis, and oxidation of two residues:

Reaction 1: isocyanate → υ_1_α-residue + (1 − υ_1_)gas (6)

Reaction 2: urethane → υ_1_ isocyanate segment +(1 − υ_2_)gas (7)

Reaction 3: isocyanate segment → υ_3_β-residue + (1 − υ_3_)gas (8)

Reaction 4: α-residue → γ-residue + (1 − υ_4_)gas (9)

Reaction 5: β-residue → δ-residue + (1 − υ_5_)gas (10)

Moreover, *ω*_I_, *ω*_UB_, *ω*_IS_, *ω*_α_, and *ω*_β_ are settled as the reaction rates for Equations (6)–(10), respectively. So the mass variations could be obtained:

*MLR*_I_ = −*ω*_I_ (11)

*MLR*_UB_ = −*ω*_UB_ (12)

*MLR*_IS_ = −*ω*_IS_ + υ_2_*ω*_UB_ (13)

*MLR*_α-r_ = −*ω*_α_ + υ_1_*ω*_I_ (14)

*MLR*_β-r_ = −*ω*_β_ + υ_3_*ω*_S_ (15)

where *MLR*_I_, *MLR*_U_, *MLR*_IS_, *MLR*_α-r_, and *MLR*_β-r_ are the mass variations of isocyanates, UB, ISs, α-residue, and β-residue, respectively.

Then the total MLR could be expressed as:

*MLR* = *MLR*_I_ + *MLR*_UB_ + *MLR*_IS_ + *MLR*_α-r_ + *MLR*_β-r_ = (υ_1_ − 1)*ω*_I_ + (υ_2_ − 1)*ω*_UB_ + (υ_3_ − 1)*ω*_IS_ − *ω*_α_ − *ω*_β_ (16)

**Figure 3:**
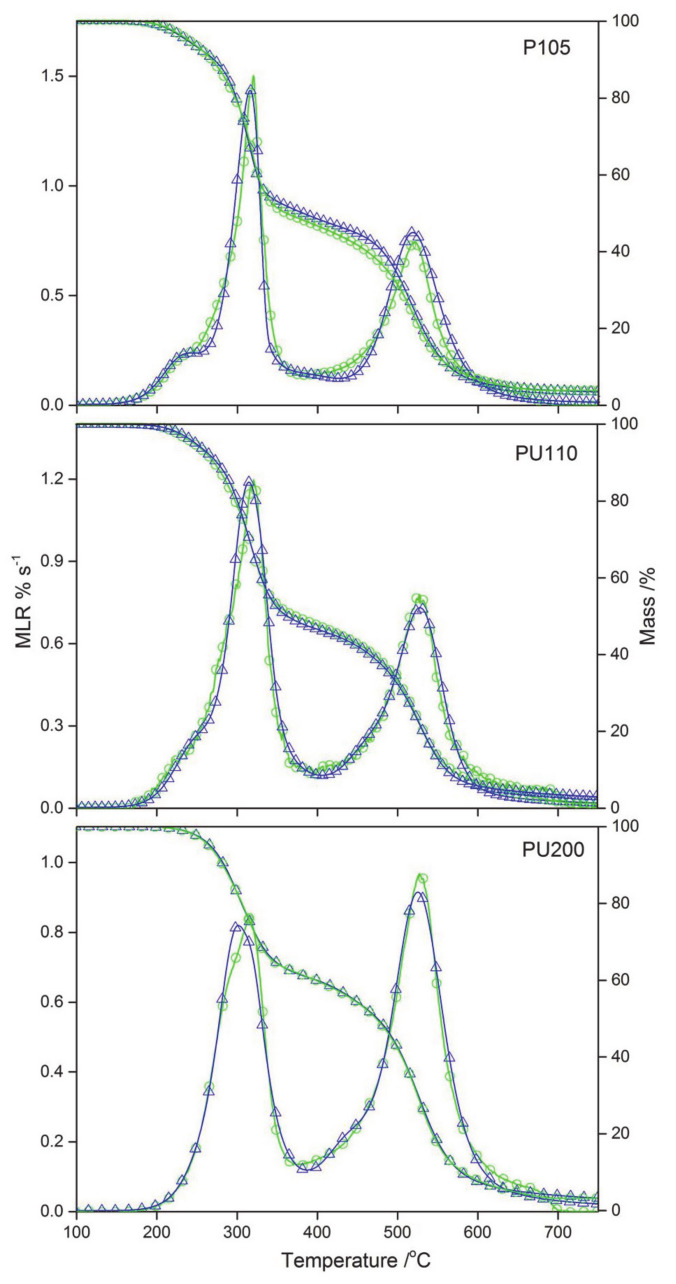
Experimental data (blue) and predicted results (green) of P105, 110, and 200 degradations in the air atmosphere at 10 °C min^−1^ heating rate.

The kinetic parameters of five sub-reactions are obtained as displayed in Table 4. In Figure 3, a better matching between experimental data and predicted values could be obtained, which also validates the accuracy of GA calculation. The Kissinger method could only obtain the active energies at two peak temperatures around 320 and 540 °C, whose results are consistent with reaction No. 4 in the GA method. This validates the accuracy of GA application in PU degradation kinetics. For other sub-reactions Nos. 1, 2, 3, and 5, different isocyanate content would have few effects. The isocyanate index would only have great influence on reaction No. 4, oxidation of the residue generated by redundant isocyanate segment.

From Table 3 and Table 4, we could find that the variations of Kissinger and GA calculation results (No. 4 reaction) keep consistent when the isocyanate ratio changed. There still some errors between the two methods, although they are not very large, 15 kJ mol^−1^ at most. We think the differential could be accepted and understood the reason that we only concluded five-step reactions during GA modeling, but there are lots of reactions during the degradation process. No.4 reaction is the dominant one at around 540 °C, and Kissinger value is the result of No.4 and other small reactions superposition.

As shown in Table 4, the reaction order parameters by the genetic algorithm are partly larger than 3. It should be noted that in the thermal analysis area, the reaction order is usually between 1 and 3, but not always. In addition, this reaction order can also change according to the reaction mechanism function. Reaction order larger than 3 and lower than 1 cases can also be found in other pyrolysis literatures [19,20,21]. The kinetics results between Kissinger method (model free) and genetic algorithm method have inconsistent results because model free method is the apparent kinetics result and the genetic algorithm result is step reaction results. The genetic algorithm result in this paper makes more sense than traditional model free method for the complex reaction shown in this paper. So by this paper we think genetic algorithm provides a new idea, that is, it could be used to separate sub-reactions from a complex reaction, and analyze each sub-reaction individually without the influence of other reactions, which is the experimental apparatus could not reach.

### 3.3. Cone Calorimeter Testing

**Figure 4:**
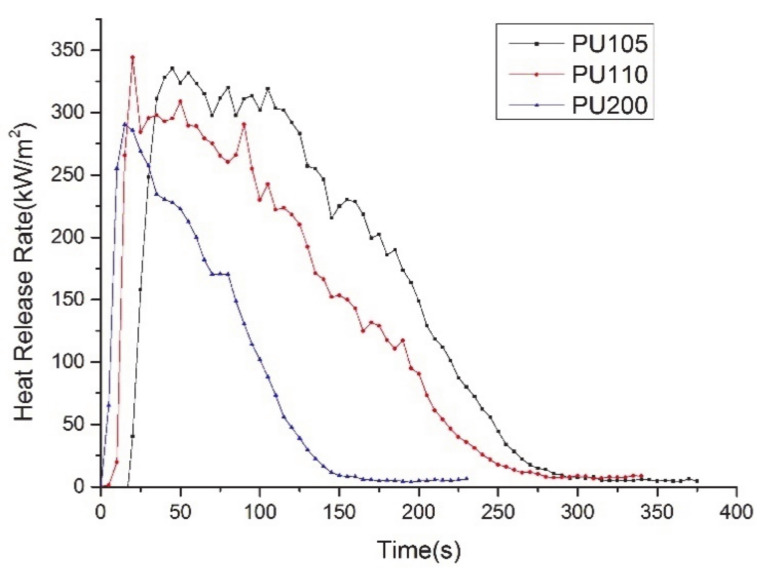
Heat release rate of PU 105, PU110, and PU200.

**Figure 5:**
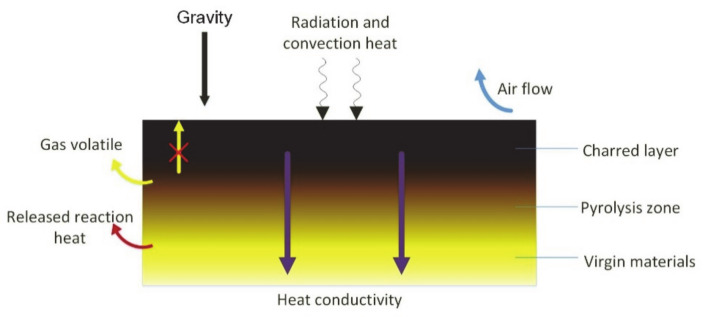
Cone calorimeter heated process of polyurethane under radiation.

Figure 4 shows the heat release rate of three materials. The process of polyurethane burning is illustrated in Figure 5, which shows the effects of the compact charred layer. HRR can reflect material’s fire hazard in the process of combustion. In addition, pkHRR is often regarded as one of the most important parts to assess the fire risk of polymer. The higher value of HRR or pkHRR is, the more heat feedback to polymer surface is. This process can make the polymer generate more volatile combustibles, which can accelerate the propagation of flame. Above all, a larger value HRR means a greater danger during a fire disaster.

As shown by the heat release rate curve of PU105, when t = 20 s, the test specimen began to release heat. In addition, at t = 45 s, it reached the pkHRR 335.38 kW/m^2^. After that, the curve showed a gradual decrease tendency until t = 125 s, during which the average value was 310 kW/m^2^. Then HRR curve of PU105 decreased sharply, and it reduced to 4.6 kW/m^2^ when t = 260 s. It showed lots of fluctuations in the latter stage. Initially, the foam was ignited by externally applied radiation, and the surface began to burn strongly. Then the HRR value rose sharply. Meanwhile, with the effects of superficial charring, HRR value began to degrease gradually after reaching pkHRR. However, burning zone of RPU began to be thicker with the process of burning. Once the internal stress of charring zone increased and eventually made the carbon layer rupture, virgin material would expose to the flame. This resulted that HRR curve increased again. This is the reason why HRR curve behaved some fluctuations during the entire test.

Different from PU105, the heat release curve of PU110 was ingited at t = 5 s and began to increase rapidly. The HRR reached the maximum at t = 20 s sharply, and the maximum value was 344.10 kW/m^2^. It maintained 300 kW/m^2^ or so at t = 20–50 s with small fluctuation. After that, the HRR decreased sharply and kept 8.77 kW/m^2^ eventually. The mass residue of PU110 was 26.71%. The total heat release maintained constant, 44.18 MJ/m^2^, after 200 s. PU200 was ignited at t = 5 s and obtained maximum HRR, 290.50 kW/m^2^, at t = 15 s. Compared with the two former materials, time to ignition and time to pkHRR were the shortest, and pkHRR was the least. Corresponding mass loss and total heat release curve revealed that the mass residue was 33.64% and total heat release was 22.09 MJ/m^2^, which was far less than the first two materials.

Furthermore, the burning time increased as the isocyanate index decreased. It is worth noting that the HRR degrade gradient increased with the increase of the isocyanate index. The HRR degradation gradient of PU105 was very small, which may be caused by more durable combustible gas during degradation. However, PUF with a high isocyanate index produced less combustible gas and burned for a shorter time.

In order to assess the safety performance of three materials thoroughly, we also contrasted THR. We could find that THRs of PU105, PU110, and PU200 were 53.1 MJ/m^2^, 44.2 MJ/m^2,^ and 22.1 MJ/m^2^ at the ending of burning in Figure 4. The increased dosage of isocyanate inversely decreased the released heat during burning. This phenomenon could be explained by the formation of an expandable carbon layer. Carbon residue after cone testing could be used as evidence that an expandable carbon layer formed. The carbon residue of PU200 was considerably larger than PU105 and PU110. It eventually remained at 33.64%. Polyurethane foam with more isocyanate generated more carbon layers to slow down the burning rate, which process is illustrated in Figure 5.

**Figure 6:**
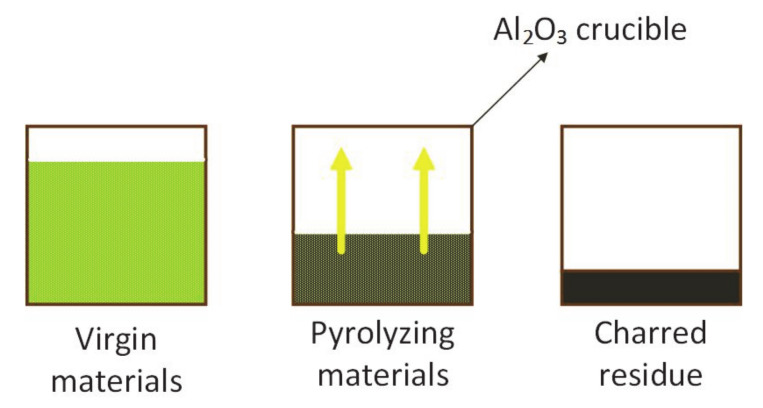
DSC testing process of polyurethane with charring volume decreasing.

For better exploring the influence of isocyanate components on total heat release and flame generation thoroughly, three materials were selected to conduct DSC testing with the heating rate of 10, 15, and 20 K/min, respectively, the total heat release has been listed in Table 5. The released heat is so tremendous that polyurethane foam materials can be ignited immediately during the pyrolysis process. In addition, this causes the formation of flame. DSC analysis results show that the released heat per unit mass rises as isocyanate index increases. Nonetheless, this phenomenon is totally different with above CONE conclusions. CONE experiments reveal that the total released heat decreases with isocyanate index increasing. This completely opposite conclusion is caused by diverse sample state of DSC and CONE. Grinded polyurethane powders were used in DSC tests, as illustrated in Figure 6, compared with a block of polyurethane foam in CONE without any grinding processes. During the reaction of CONE, the sample is heated by the radiation heater, which leads formation of carbon layer at the combustible surface which can isolate oxygen and heating for further reaction. As isocyanate index increases, the carbon layer becomes denser, which can cause less released heat. However, during DSC test, grinded polyurethane powders are employed and can not transform to dense carbon layer after pyrolysis. The difference of the sample shape leads to the difference of the two calorimetry trends.

## 4. Conclusions

In this work, three PUs with different isocyanate indexes, P105, P110, and P200, were manufactured. In addition, a series of TG experiments were carried out to compare their thermal stability. The following conclusions are presented.

1. With the increase of the isocyanate index, the initial degradation temperature of PUs would increase. The initial temperature of P200 is higher than P105 and P110, obviously. The mass loss of the degradation part would decrease, and the oxidative part would increase.
2. Calculation by Kissinger method shows that in both degradation stages, P105 shows the highest activation energy, and P110 is the lowest activation energy, which means P110 is much easier to degrade than P105.
3. Considering the PU degradation mechanism in air, its degradation process was divided into five steps. The calculations kinetic results by GA method were consistent with those by Kissinger method, which verified the accuracy of this new kinetic method. By calculation and reaction separation using GA, we find that isocyanate contents have a great influence on the oxidation of the residue generated by redundant isocyanate.
4. The variation of total heat release with isocyanate index by CONE testing was absolutely different from DSC results. This was caused by the different sample states of two groups of experiments. Whole polyurethane foam was used in CONE tests, and a retardant carbon layer was generated later. Whereas ground powders were adopted in DSC, and only the residue with no retardant effects was left.

## Figures and Tables

**Table 1:** The reagent used in the preparation of PUFs.

Sample	P105/G	P110/G	P200/G
Polyether polyol	143	135	59
Isocyanate	150	150	150
Dibutyltin dilaurate	0.5	0.5	0.5
Silicone oil	2	2	2
Distilled water	2	2	2
A-33	1	1	1
Triethanolamine	3	3	3
Isocyanate index	1.05	1.1	2.0

**Table 2:** Pyrolysis parameters of PUs with different heating rates in the air atmosphere.

Heating Rate /°C/Min	Degradation Phase			Oxidative Phase		
	T_o_/°C	T_p_/°C	Mass Loss/%	T_o_/°C	T_p_/°C	Mass Loss/%
*P105*						
10	146.95	320.93	54.14	382.64	526.54	45.86
15	150.50	326.12	54.47	397.19	533.69	45.53
20	152.94	329.29	54.86	404.36	540.97	45.14
*P110*						
10	162.88	319.27	51.95	398.23	526.66	48.05
15	171.65	326.98	51.39	420.33	540.34	48.61
20	180.61	331.64	51.61	429.36	548.55	48.39
*P200*						
10	196.00	313.58	39.10	395.43	526.79	60.90
15	199.84	320.66	39.21	409.33	539.40	60.79
20	202.99	325.18	40.54	419.64	546.69	59.46

**Table 3:** Kinetic calculation results by the Kissinger method.

	Activation Energy /Kj Mol^−1^	
PU	Degradation Phase	Oxidative Phase
105	235.20	246.57
110	155.76	158.17
200	163.77	174.59

**Table 4:** Kinetic triplets of PUs calculated by GA method.

Reaction No.	Kinetic Parameter	P105	P110	P200
1	*A*/log_10_(s^−1^)	9.83	10.17	10.45
	*E*/kJ/mol	111.34	112.71	115.34
	*n*	2.33	2.31	3.16
2	*A*/log_10_(s^−1^)	13.50	13.55	12.91
	*E*/kJ/mol	175.68	174.32	163.29
	*n*	0.75	1.31	1.72
3	*A*/log_10_(s^−1^)	9.43	9.42	10.65
	*E*/kJ/mol	120.54	123.31	128.56
	*n*	4.31	5.84	5.54
4	*A*/log_10_(s^−1^)	14.98	14.98	14.94
	*E*/kJ/mol	229.04	150.81	173.02
	*n*	4.60	2.58	3.85
5	*A*/log_10_(s^−1^)	13.59	13.59	13.65
	*E*/kJ/mol	230.23	237.54	236.33
	*n*	2.32	1.52	1.81

**Table 5:** Heat release during DSC testing kW m^−2^.

Heating Rates/(K min^−1^)	PU105	PU110	PU200
10	6760	7130	7329
15	6360	7023	7294
20	6030	6608	7363

## Data Availability

Not applicable.

## Nomenclature

a	mass coefficient
A	pre-exponential factor
DTG	derivative thermogravimetry
EPS	expandable polystyrene
E	activation energy
FTIR	Fourier transform infrared
g	gene
GA	genetic algorithm
IS	isocyanate segment
MLR	mass loss rate
PU	polyurethane
PUs	polyurethanes
PUF	polyurethane foam
Py-GC/MS	pyrolysis-gas chromatography/mass spectrometry
RPUs	rigid polyurethanes
t	time
T	temperature
TG	thermogravimetry
TG-MS	TG-mass spectrometry
T_p_	peak temperature
UB	urethane bond
XPS	extruded polystyrene
α	conversion percent
β	heating rate
